# Lessons from the Fourth Metronomic and Anti-angiogenic Therapy Meeting, 24–25 June 2014, Milan

**DOI:** 10.3332/ecancer.2014.463

**Published:** 2014-09-09

**Authors:** Gauthier Bouche, Nicolas André, Shripad Banavali, Frank Berthold, Alfredo Berruti, Guido Bocci, Giovanni Brandi, Ugo Cavallaro, Saviero Cinieri, Marco Colleoni, Giuseppe Curigliano, Teresa Di Desidero, Alexandru Eniu, Nicola Fazio, Robert Kerbel, Lisa Hutchinson, Urszula Ledzewicz, Elisabetta Munzone, Eddy Pasquier, O Graciela Scharovsky, Yuval Shaked, Jaroslav Štěrba, Martin Villalba, Francesco Bertolini

**Affiliations:** 1 Anticancer Fund, Strombeek-Bever B1853, Belgium; 2 Metronomics Global Health Initiative; Aix Marseille Université, Inserm, CRO2 UMR_S 911; & Paediatric Haematology and Oncology Department, Children’s Hospital of La Timone, Marseille 13005, France; 3 Tata Memorial Centre, Mumbai 400012, India; 4 Department of Paediatric Oncology, University of Cologne D50924, Germany; 5 Department of Medical and Surgical Specialties, Radiological Sciences and Public Health, University of Brescia, Azienda Ospedaliera Spedali Civili, Brescia 25123, Italy; 6 Division of Pharmacology, Department of Clinical and Experimental Medicine, University of Pisa, via Roma 55, Pisa 56126, Italy; 7 Department of Experimental, Diagnostic and Specialty Medicine University Hospital S. Orsola-Malpighi Bologna, 40138, Italy; 8 Molecular Medicine Programme, European Institute of Oncology, Milan 20141, Italy; 9 Medical Oncology Department, Brindisi 72100, Italy; 10 Division of Medical Senology, European Institute of Oncology, European Institute of Oncology, Milan 20141, Italy; 11 Division of Experimental Therapeutics, European Institute of Oncology, Milan 20141, Italy; 12 Cancer Institute ‘I. Chiricuta’, Cluj-Napoca 400015, Romania; 13 Unit of Gastrointestinal Medical Oncology and Neuroendocrine Unit, European Institute of Oncology, Milan 20141, Italy; 14 Sunnybrook Research Institute, Sunnybrook Health Sciences Centre, University of Toronto M4N 3M5, Canada; 15 Nature Reviews Clinical Oncology, London N1 9XW, UK; 16 Department of Mathematics and Statistics, Southern Illinois University, Edwardsville, IL 62026, USA; 17 Division of Medical Senology, European Institute of Oncology, Milan 20141, Italy; 18 Tumour Biology and Targeting Programme, Children’s Cancer Institute Australia, Lowy Cancer Research Centre, University of New South Wales, Randwick 2031, Australia; Metronomics Global Health Initiative, Marseille 13005, France; & Centre for Research in Oncobiology and Oncopharmacology, INSERM UMR911, Marseille 13005, France; 19 Jefa Sección Oncología Experimental, Instituto de Genética Experimental, Facultad de Ciencias Médicas, Universidad Nacional de Rosario, 2000, Argentina; 20 Rappaport Faculty of Medicine, Technion, Haifa 31096, Israel; 21 Department of Pediatric Oncology, Masaryk University School of Medicine and University Hospital, Brno, Cernopolni 9 Brno 613 00, Czech Republic; 22 INSERM U1040, Université de Montpellier 1, UFR Médecine, Montpellier 34295, France & Institute for Regenerative Medicine and Biotherapy (IRMB), CHU Montpellier, Montpellier 34295, France; 23 Laboratory of Haematology-Oncology, European Institute of Oncology, Milan 20141, Italy

**Keywords:** metronomic chemotherapy, cancer, drug repurposing, anti-angiogenesis, pharmacoeconomics, adult, child

## Abstract

The Fourth Metronomic and Anti-angiogenic Therapy Meeting was held in Milan 24–25 June 2014. The meeting was a true translational meeting where researchers and clinicians shared their results, experiences, and insights in order to continue gathering useful evidence on metronomic approaches. Several speakers emphasised that exact mechanisms of action, best timing, and optimal dosage are still not well understood and that the field would learn a lot from ancillary studies performed during the clinical trials of metronomic chemotherapies. From the pre-clinical side, new research findings indicate additional possible mechanisms of actions of metronomic schedule on the immune and blood vessel compartments of the tumour micro-environment. New clinical results of metronomic chemotherapy were presented in particular in paediatric cancers [especially neuroblastoma and central nervous system (CNS) tumours], in angiosarcoma (together with beta-blockers), in hepatocellular carcinoma, in prostate cancer, and in breast cancer. The use of repurposed drugs such as metformin, celecoxib, or valproic acid in the metronomic regimen was reported and highlighted the potential of other candidate drugs to be repurposed. The clinical experiences from low- and middle-income countries with affordable regimens gave very encouraging results which will allow more patients to be effectively treated in economies where new drugs are not accessible. Looking at the impact of metronomic approaches that have been shown to be effective, it was admitted that those approaches were rarely used in clinical practice, in part because of the absence of commercial interest for companies. However, performing well-designed clinical trials of metronomic and repurposing approaches demonstrating substantial improvement, especially in populations with the greatest unmet needs, may be an easier solution than addressing the financial issue. Metronomics should always be seen as a chance to come up with new innovative affordable approaches and not as a cheap rescue strategy.

## Introduction

Prof. Francesco Bertolini (Laboratory of Haematology-Oncology, European Institute of Oncology, Milan, Italy), organiser of the conference (which was held at the European Institute of Oncology) warmly welcomed all attendees and started by reminding the audience that metronomic chemotherapy as well as repurposed drugs are not only promising approaches in many different cancer types and populations, but they represent a major opportunity for National Health Services in high-income as well as low- and middle-income countries (LMICs) to save money without compromising on efficacy and safety. The cost of recently approved anticancer drugs is known to have become unaffordable even for the richest countries in the world. He illustrated this situation by reporting that pertuzumab and TDM-1, two drugs which have shown efficacy in phase III trials in breast cancer, will not be reimbursed by the Italian health service system because of their cost. Reimbursing these drugs would only increase the 1.8 billion deficit accumulated until now.

## Session 1: The landscape of the field

Robert Kerbel (Sunnybrook Research Institute, Sunnybrook Health Sciences Centre, University of Toronto) gave an overview of his experience in the field since the two seminal papers published in 2000, one from his group [1] and the other from Dr Folkman’s group [2]. At that time, the impressive results—in several models, including transplanted primary human neuroblastoma (NB) xenografts, and transplanted primary mouse tumours, including lung cancer and melanoma—indicated that frequent regular administration of low-dose chemotherapy was superior to maximum tolerated dose chemotherapy in suppressing tumour growth and increasing survival of the mice. In an accompanying editorial, commenting on the study by Kerbel’s group, and also on the study from Folkman’s group, Hanahan and colleagues coined the term ‘metronomic’ to describe this way of administering chemotherapy [3]. As of June 2014 Kerbel was able to identify approximately 90 clinical trials designed to evaluate the safety and efficacy of metronomic chemotherapy in a variety of cancers. This information is summarised under the heading of ‘metronomic chemotherapy’ in www.clinicaltrials.gov. Most of these trials are small phase II trials, the majority of which are non-randomised, but there are also a number of randomised phase III clinical trials. This information highlighted the fact that metronomic chemotherapy largely remains a niche treatment concept in oncology, the reasons for which were discussed in depth at this meeting. Positive phase III results could significantly change this circumstance (see below). Coming back to initial observations, stemming from his own work and that of others, Kerbel listed the main mechanisms of action described in the literature to account for the antitumour effects of metronomic chemotherapy. These include:

- targeting endothelial cells of the tumour neovasculature
- inhibiting mobilisation or directly targeting pro-angiogenic bone marrow derived cell populations (BMDCs)
- suppressing hypoxia-inducible factor1-apha (HIF1-alpha)
- affecting tumour cells directly, possibly including cancer stem cells
- stimulating the immune system.

These effects can depend heavily on the nature of the chemotherapy drug studied.

Kerbel went on to highlight recent findings from his group, showing the promising impact of various metronomic chemotherapy regimens when treating mice with advanced metastatic disease. This is in contrast to certain other therapies, including targeted anti-angiogenic drugs. For example, when testing sunitinib in mice with advanced metastatic breast cancer, the drug showed no activity in terms of prolonging survival, even when combined with standard chemotherapy. This correlated with previous randomised phase III clinical trials of sunitinib alone or with chemotherapy. In contrast, treating mice with established primary tumours indicated that the drug was active in this setting. The opposite pattern was observed when treating mice with the same tumour line, using metronomic chemotherapy, e.g., Tegafur-uracil (UFT) plus cyclophosphamide-two oral chemotherapy drugs. Mice with metastatic disease experienced prolonged survival, whereas this was not the case when mice with primary tumours were treated with the same regimen. This implies that metronomic chemotherapy may not work in certain circumstances by inhibiting angiogenesis. The results also highlight the benefits of using mouse models of metastatic disease for investigational therapeutics, in contrast to the more traditional approach of using mice that only have primary tumours.

Kerbel then summarised some recent completed phase III trials that were designed to evaluate the safety and efficacy of metronomic chemotherapy regimens. One trial, called CAIRO3, run by the Dutch Colorectal Cancer Group, evaluated a continuous lower-dose daily oral capecitabine regimen, combined with bevacizumab, as a follow-up maintenance therapy in first-line metastatic colorectal cancer patients, after they had received a standard regimen of chemotherapy involving capecitabine given in a more conventional schedule (two weeks on/ one week off, using higher daily doses), combined with oxaliplatin and bevacizumab (CAPOX-B). If patients showed a response to the initial upfront therapy they were randomised to the maintenance regimen or observation only. When patients relapsed in either arm, they were then retreated with the original upfront CAPOX-B regimen. The patients in the maintenance treatment arm showed a significant benefit in progression-free survival (PFS), especially after the maintenance treatment, but also after retreatment with CAPOX-B. Subgroup analysis also indicated that overall survival benefit in certain patients, e.g., those whose primary tumour had been resected, and had synchronous metastases at the time of resection. Another completed phase III trial was undertaken in Switzerland, in metastatic breast cancer patients as first-line therapy (called SAKK-24-09). However, the number of patients enrolled in this trial, made it more like a randomised phase II trial. The experimental arm consisted of metronomic cyclophosphamide plus capecitabine and bevacizumab whereas the standard arm consisted of weekly paclitaxel plus bevacizumab. The efficacy results were virtually identical between the two arms, which is encouraging. However, toxicity was actually greater in the experimental arm compared to the standard and since toxicity was the primary endpoint, this trial was considered negative. Kerbel pointed out that the toxicity seen in the standard arm was less than in the corresponding E2100 phase III trial evaluating the weekly paclitaxel plus bevacizumab regimen, amd the toxicity in the experimental arm was greater than previously observed in a non-randomised phase II trial evaluating the metronomic cyclophosphamide plus UFT regimen in combination with bevacizumab in metastatic breast cancer patients. Thus, taken together, these two trials provide a rationale for continuing clinical studies of metronomic chemotherapy. Kerbel concluded by highlighting open questions on the optimal dose of metronomic chemotherapy, the optimal treatment settings, optimal combination treatments, and a possible requirement or benefit of individualised dosing and scheduling, possibly using low-grade toxicity as a guide. He also stressed the need to further define the cellular and molecular mechanisms responsible for the antitumour effects of metronomic chemotherapy regimens.

Lisa Hutchinson, Chief Editor of *Nature Reviews Clinical Oncology*, was invited to explain the reasons behind the journal’s interest in the topic of metronomic chemotherapy, which includes publishing two reviews—the first one in 2010 [4] and a very recent update in 2014 [5], published online a couple of weeks before the Milan meeting. Part of the motivation for commissioning an update of the first article published in 2010 was that this was a highly cited article and thus was popular among the journal’s readership, as well as the need to cover topics that are under-highlighted in the literature. The potential of metronomic chemotherapy for the medical oncology community in a broader context was also discussed in a recent editorial. She continued her talk by emphasising the advantages of metronomic chemotherapy, drug repositioning, the mechanisms of action of metronomics, the success of this therapy approach, and the key reasons behind the general scepticism of the potential of metronomic chemotherapy within the cancer community.

Importantly, she elaborated on the editorial team of *Nature Reviews Clinical Oncology*, its editorial processes, and those of the Nature Review’s journals. She highlighted the professionalism and expertise of the team leading the journal, as well as emphasising that the journal is editorially independent and also the importance of staying in touch with the field by attending conferences, such as this one.

Yuval Shaked (Rappaport Faculty of Medicine at the Technion, Haifa, Israel) had the task of describing preclinical new hypotheses to explain the therapeutic effect of metronomic chemotherapy. The first question he addressed was whether the tumour environment is changed during maximum tolerated dose (MTD) chemotherapy regimen when compared to metronomic chemotherapy regimen. Using non-tumour bearing mice treated with bolus dose of paclitaxel (but not gemcitabine), he showed that there is an induction in endothelial progenitor cells (EPCs) in the blood, an effect which was ‘blunted’ by an upfront treatment with an anti-angiogenic drug [6]. Factors such as granulocyte colony-stimulating factor (GCSF) and stromal cell derived factor (SDF1) but not vascular endothelial growth factor (VEGF) were also induced in the plasma in response to paclitaxel chemotherapy but not in response to gemcitabine. He concluded that the host, in response to acute MTD regimen, generates a storm of growth factors which in turn may promote tumour re-growth and angiogenesis. He also showed that these host effects following MTD chemotherapy can also lead to tumour cell dissemination from the primary tumour and acceleration of metastasis. He mentioned that experiments from Bertolini published already in 2003 [7] demonstrated similar results when focusing on EPCs, distinguishing between the levels of CEPs following MTD cyclophosphamide and metronomic cyclophosphamide.

In a recent study, Shaked’s lab found that similar to EPCs there is also an increase in myeloid derived suppressor cells (MDSCs) expressing the surface markers GR1+/CD11b+ in the blood of non-tumour bearing mice following treatment with weekly gemcitabine (representing MTD regimen) when compared to daily gemcitabine (representing metronomic regimen). He showed that the rebound in MDSC levels following MTD gemcitabine can be blocked by a switching the treatment to metronomic gemcitabine, thus keeping low levels of MDSCs during the treatment period. This ‘chemo-switch’ regimen could explain the recent results from his lab in which mice bearing an orthotopic pancreatic tumour model extended the survival, when they were treated with the chemo-switch regimen, confirming the superiority of this regimen initially proposed by Kerbel and Hanahan in 2005 [8, 9]. In this new experiment the best approach to control tumour growth was the chemo switch regimen which not only reduced the tumour growth and controlled it, but also it inhibited metastasis and increased mouse survival. In comparison, the MTD regimen resulted in an initial complete response following the therapy followed by a rapid regrowth and increased metastasis, whereas the pure metronomic regimen resulted in a very slow growth of the tumour with minimal metastasis lesions detected [10]. However, Shaked stressed that focusing on one or two cell types does not allow a systemic view of the changes occurred at the tumour microenvironment following therapy. Specifically, the subtypes of cells which may impact the tumour microenvironment following therapy such T-regs, fibroblasts, tumour associated macrophages (TAM), and others. He therefore suggested that using a Time of Flight Mass Cytometry (CyTOF) technology and Spade analysis could nicely represent many different cell population colonising the tumour microenvironment, which can be tested. This work is still in its infancy and need further replication and validation before it can be published.

## Session 2: Breast cancer, paediatric cancer, and models

Marco Colleoni (Division of Medical Senology, European Institute of Oncology, Milan, Italy) gave a comprehensive overview on the metronomic experience in breast cancer. He described the trials performed until now as quite empirical when compared to targeted approaches, with low numbers, and were rarely randomised.

Overall, about 15% of patients with metastatic breast cancer derived a long term benefit (for more than 12 months) of metronomic approaches. His group had performed several trials in the metastatic setting and results indicate a clinical benefit rate (CBR) of 60% or more with various combinations and in various population. Experience of metronomic chemotherapy with targeted agents are also encouraging, be it with trastuzumab [11] or with bevacizumab and/or trastuzumab in the BEX/BEXET trial, even though some coronary heart disease (CHD) was observed in patients treated with bevacizumab and trastuzumab.

In the adjuvant setting, similar activity between an MTD regimen and a metronomic regimen have been observed as in the Japanese trial reported in 2009 by Watanabe [12]. The IBCSG 22-00 trial has just completed recruitment and randomised breast cancer patients [70% of triple-negative breast cancer (TNBC)] between CM maintenance and observation and first results should be released in spring 2015.

In the neoadjuvant setting, a randomised trial did not show any difference between the standard arm and the metronomic arm in terms of response [13]. Other experience adding metronomic protocol to standard neoadjuvant chemotherapy regimen showed interesting pathologic complete response (PCR) rates but in limited numbers, and the absence of randomisation precluded making any definitive conclusion.

Overall, it is clear from the breast cancer experience that metronomic chemotherapy has shown activity and is able to control the disease in a large proportion of patients while limiting toxicity and impact on quality of life (QOL). For the future, the design of the trials could be improved with better patient selection, better rationale for the choice of the combination, and choice of the most clinically relevant endpoints.

Elisabetta Munzone (Division of Medical Senology, European Institute of Oncology, Milan, Italy) continued the discussion about breast cancer with a view on the future. New possible mechanisms of actions including an effect on the immunity and especially a decrease in the number of Tregs, or an inhibition of the function of Tregs. A 2012 paper by Ge and Domschke [14] showed in metastatic breast cancer patients that Tregs were only transiently depleted during metronomic cyclophosphamide but tumour reactive T-cells were increased for the whole treatment period, which indicates a more complex and potent immunomodulation of metronomic cyclophosphamide.

New directions in designing metronomic chemotherapy trial should be on looking at biomarkers and reliable surrogate markers of clinical benefit. The neoadjuvant and adjuvant settings seemed also to be interesting settings for positioning metronomic chemotherapy. She listed some of the most interesting adjuvant trials such as the ABCDE trial (NCT00925652) where two of the four arms will receive metronomic cyclophosphamide with bevacizumab (plus diet intervention +/- exercise), the SYSUCC-001 trial (NCT01112826) in TNBC patients where the intervention group receive one year of metronomic capecitabine maintenance after standard adjuvant therapy, and the IBCSG 22-00 trial (NCT00022516) testing for cyclophosphamide-methotrexate maintenance after standard adjuvant therapy in hormone receptor negative patients.

In the neoadjuvant/post-neoadjuvant setting, she identified seven trials. She also highlighted that Masuda recently reported [15] encouraging results with metronomic paclitaxel/cyclophosphamide/capecitabine followed by Fluorouracil (5FU), epirubicin and cyclophosphamide (FEC) chemotherapy. This regimen was associated with a high PCR rate and low toxicity in TNBC patients.

In the metastatic setting, she identified 17 ongoing trials with metronomic regimen (ten with capecitabine and seven with other drugs), including the VEX trial (EUDRACT 2010-024266-21) evaluating the role of the combination of vinorelbine, cyclophosphamide, and capecitabine administered metronomically. The trial had already recruited 59 of the 146 metastatic patients planned at the time of her presentation, and preliminary results were presented.

She concluded by looking at the breast cancer landscape and suggesting places where metronomic chemotherapy could be positioned. The role of metronomic chemotherapy seems particularly promising in the neoadjuvant and maintenance settings.

Continuing in breast cancer, Giuseppe Curigliano (Division of Experimental Therapeutics, Istituto Europeo di Oncologia, Milano, Italy) presented results from a trial in patients with recurrent inflammatory breast cancer (IBC) with lymphangitic spread to the chest wall using bevacizumab before or concurrent to vinorelbine and capecitabine. Lymphangiogenesis is common in IBC; it may contribute to IBC spread. Higher expression of lymphangiogenic factors (VEGF-C, VEGF-D, VEGFR-3, Prox-1, and fibroblast growth factor 2) are detected in IBC than in non-IBC tumour samples. Several treatments targeting lymphangiogenesis have been investigated in cancer in general, but not specifically in IBC. Targeting lymphangiogenesis through the VEGF-C/VEGF-D/VEGFR-3 signalling system would be a reasonable therapeutic approach for IBC, although it will need to be further examined in both preclinical and clinical studies.

They used IBC as a model disease to investigate biological changes associated with an antiangiogenic agent as bevacizumab. The biological study on circulating endothelial cells (CEC), circulating endothelial progenitor (CEP), and CPP as surrogate predictive biomarkers showed that at baseline, responders had significantly higher counts of a CEC subpopulation expressing VEGFR2 and of CPPs (possibly involved in vessel stabilisation). On the other hand, baseline counts of CEPs, of viable CECs, and of the inflammation-related chemokine IL-8 below the median value were associated with a significantly improved overall survival (OS). These data, if confirmed in larger series of patients, might suggest a reduced survival in patients with intense angiogenic activity (as reflected by high levels of CEP and viable CECs) and IL-8-mediated inflammatory response.

To date, no molecular feature reliably predicts the response to bevacizumab. Using DNA microarrays, they searched for multigene predictors for response in IBC with lymphangitic spread to the chest wall. They identified 160 genes that clearly separated patients that had achieved partial respone from those that had no response. A supervised clustering identified 75 genes involved in matrix remodeling (MMP1) and cell cycle regulation (CDKN2A). The response to bevacizumab seems enhanced by a pre-existing stroma remodeling environment. This signature was strongly enriched for stroma that clearly highlighted the importance of tumour-stroma crosstalk for progression of IBC.

Giovanni Brandi (Department of Experimental, Diagnostic and Specialty Medicine University Hospital S. Orsola-Malpighi Bologna, Italy) took us from patients with breast cancer to patients with hepatocellular carcinomas (HCC). In this population, systemic treatment mainly relies on sorafenib which has limited benefit and some toxicity, with approximately 30% of patients having to stop the drug because of side effects. The strong angiogenesis and the high level of endothelial progenitor cells found in HCC make metronomic chemotherapy an interesting candidate for this disease.

He had identified six trials, testing a metronomic chemotherapy regimen in HCC, three of them in combination with sorafenib. He presented data from their own experience with metronomic chemotherapy alone. In 2010, they published a dramatic response in one patient with metronomic capecitabine alone [16] and he presented two other unpublished cases with impressive response, including one with histological confirmation of complete response. Moving to the trials they performed, the first-line metronomic capecitabine was well tolerated and gave a median OS of 15.6 months which compared favourably to matched untreated controls from the ITA.LI.CA. database (of eight months) [17]. In second-line and in patients with Child-B disease, their experience also seems promising, but in comparison with historical data showed only moderate improvement in OS.

They have now designed two trials for which they are currently assessing funding opportunities. The first is a pilot study on the association of everolimus with metronomic capecitabine in HCC/OLT patients at high risk of relapse; the second is a phase III randomised trial comparing metronomic capecitabine/sorafenib to sorafenib/metronomic capecitabine in advanced HCC patients.

The two final presentations of the first day were reported experiences in paediatric cancers.

Jaroslav Štěrba (Brno School of Medicine, Brno, Czech Republic) set the scene of metronomic chemotherapy in paediatric oncology. He described four cases of patients who responded to and benefited from a metronomic approach, one patient with anaplastic astrocytoma, and three patients with medulloblastoma. He emphasised that some of the response were only seen on 18-Fluoro-deoxyglucose positron emission tomography (FDG-PET), highlighting that PET Response Criteria in Solid Tumours (PERCIST) [18] may be a better endpoint than Response Evaluation Criteria In Solid Tumours (RECIST) for metronomic chemotherapy.

He listed some of the inherent problems of paediatric oncology and emphasised the very limited number of phase I trials happening every year in children with cancer in the European Union (EU)—fewer than ten compared to more than 200 phase I trials in adult oncology per year in 2008–2013.

He reminded the audience of their results with the COMBAT (Combined Oral Metronomic Biodifferentiating Antiangiogenic Treatment) regimen, which included low-dose daily temozolomide, etoposide, celecoxib, vitamin D, fenofibrate, and retinoic acid, and the conclusion that COMBAT is a feasible, effective, and low-toxicity treatment option for patients with relapsing/refractory malignancies [19]. When looking at the long-term survivors of this regimen, we find 12 out of 13 patients with low-grade tumours, only one high-grade sarcoma, five of 22 high-risk NB, and six of 13 medulloblastoma.

Despite encouraging results in relapsed setting, attempts to bring metronomics as maintenance for very high-risk first complete remission (CR) paediatric patients are still not translated into current practice.

Frank Berthold (Department of Paediatric Oncology, University of Cologne, Germany) reported his experience with a focus on NB. He presented data from a retrospective analysis of 246 patients with high-risk NB. These patients have a very poor survival after recurrence and chemotherapy has no impact on the outcome, as shown by his data. Only patients who could undergo a second allogeneic stem cell transplantation had an increased survival, a benefit which was however still limited.

He explained that NB is a prototypical highly vascularised tumour. In addition, vessels are immature and of embryonic type in NB. Patients with advanced or high-risk NB have high VEGF and high levels of other makers of angiogenesis. This makes NB a good candidate for anti-angiogenic treatment if bevacizumab works well in *in vivo* models. The four small pilot studies in NB only included a very limited number of patients, which precluded making any conclusion. A larger European randomised trial, the BEACON trial (EUDRACT 2012-000072-42) is ongoing and should address this question.

Since NBs express high levels of cyclooxygenase-2 (COX-2) and preclinical data have confirmed the potential of COX-2 inhibitors, celecoxib has been tested in combination with other drugs in five pilot studies in NB with some responses. Following the Second Metronomic Meeting in Marseille in 2010, the METRO-NB 2012 trial is now being implemented and tested using celecoxib, cyclophosphamide, methotrexate and vinblastine in 26 patients with event-free survival (EFS) as the primary endpoint. A pilot study was first done in 15 patients to assess the feasibility and showed that some grade 3 toxicities were observed as well as some responses which allowed to move to the next phase. All children could be treated in an outpatient setting. The myelodepressive side effects resulted very likely more from the preceding chemotherapy (MTD approach) than from the current metronomic study.

Urszula Ledzewicz (Department of Mathematics and Statistics, Southern Illinois University, Edwardsville, USA) works with her team on mathematical models that describe biomedical approaches. They have analysed mathematical models to compare the three main chemotherapeutic approaches discussed during this meeting: MTD (called bang bang), metronomic chemotherapy (called singular), or Chemo-Switch (first bang-bang followed by singular). To do this, she used four different contexts: a model with a homogeneous population of cancer cells, a model with heterogeneous populations of cancer cells, a model taking into account the vasculature compartment of the tumour microenvironment, and a model taking into account the immune system interactions.

In a model of homogeneous cancer cells, an MTD approach works best, whereas the singular approach was less effective [20]. In a model of heterogeneous cancer cells, the Chemo-Switch was the most effective. In a more comprehensive model taking into account the vasculature, it was important to start with full dose (bang bang) in order to have an effect on the vasculature and then to switch to the singular control followed by a rest period. The singular part is the one responsible for the major part of the shrinking of the tumour [21].

In the last model, adding the immune system interactions, a geometrical representation called phase portrait was used to describe the model. Basically, this model has two main points of equilibrium, one with no growth and a competent immune system (the good equilibrium), and the other with strong growth and depressed immune system (the bad equilibrium). Each of them has its region of attraction called good and bad regions, respectively. In this model, the MTD approach (bang-bang control) brought the point from the bad region close to crossing the boundary with the good region. However, it was then necessary to switch to metronomic chemotherapy to be able to actually cross this boundary and go towards the good equilibrium [22].

She concluded by saying that the ideal approach to reach the good equilibrium would be a feedback dose or adaptive therapy. This would require adjusting the dose depending on the tumour growth with no need to actually eradicate the tumour. She also pointed in the future directions, she would like to work on the model of chaotic therapy proposed in collaboration with Nicolas André and Eddy Pasquier.

Summarising, the MTD therapy alone was effective in models which did not take into account neither the heterogeneity nor the tumour microenvironment (vasculature, immune system). Once any of these aspects are included into the model, the optimal protocols were MTD followed by metronomic with a time-varying, feedback-type dose.

## Session 3: Biomarkers and repositioning

Guido Bocci (University of Pisa, Italy), gave a presentation about biomarkers. Starting with the definition from Marc Buyse and colleagues in their 2010 review [23], he showed that validated predictive biomarkers are very few in the oncology field (e.g., K-ras mutation predictive of lack of activity of cetuximab and panitumumab in colon cancer). He also broadened the view of biomarkers, mentioning the different types of biomarkers and that some, like imaging methods, were usually under-highlighted. To him, the main challenges for biomarkers are the following:

- the availability of the biological material
- the need of multiple testing methods
- the quality of a good assay: quick, reliable, inexpensive, and easy
- the validation part
- the need of cooperation between industry and academia.

Coming back to metronomic chemotherapy, he underlined as important that maybe ‘simple’ pharmacokinetic (PK) markers would be interesting. For instance, for the classical 50 mg cyclophosphamide, there is no PK study to support this regimen. Why is this important then? Because if one believes in the concept of metronomic chemotherapy, it is important to know and reach an optimal, constant concentration of the drug, or its active metabolites over time, which he called concentration at a steady state (Css), that is effective. Further important information that the PK study would give on metronomic chemotherapy is the possibility to find the correct dose reduction which is required compared to conventional regimen, since most doses used are rather empirical with no consensus on the best dose. Moreover, PK knowledge could add precious information on any possible PK interactions (positive or negative) with other drugs, such as tyrosine kinase inhibitors or therapeutic antibodies.

He then cited examples of PK studies performed with metronomic regimen and the wealth of information provided by these studies, such as the studies reported by Stempak in 2006 with vinorelbine or cyclophosphamide in paediatric tumours [24], Allegrini in 2008 with metronomic irinotecan in colorectal cancer [25], and Allegrini in 2012 [26] with metronomic UFT in gastrointestinal cancers. In the latter, the maximal concentration (Cmax) of 5-FU (the active metabolite of UFT) in plasma was higher in patients with stable disease than in patients with progressive disease. The 5-FU Cmax higher than 0.5 µg/ml after the very first administration was predictive of a survival benefit. The MOMA study, a phase II trial of 222 colorectal cancer patients, randomised between bevacizumab alone and bevacizumab + capecitabine metronomic includes an extensive PK study and will provide further information. Regarding the pharmacogenomic markers of metronomic chemotherapy, he showed that some genes, such as TSP-1, increase their expression in a time and concentration-dependent manner in treated cancer patients [25]. Moreover, CD133 gene expression has been proposed as a potential pharmacogenomic marker of metronomic UFT [26]. The personalisation of metronomic chemotherapy could be obtained through a pharmacogentic analysis of candidate patients. Indeed, a recent pilot study suggested that VEGF SNPs (i.e., VEGF-634C/G) may be predictive of PFS benefits [27].

From a pharmacodynamics point of view, the most used—but not validated—biomarker of benefit from metronomic chemotherapy is the level of plasma VEGF [28]. This was also clearly observed in a metastatic model of spontaneous canine tumours treated with metronomic cyclophosphamide where high plasma VEGF was associated with lower PFS [29].

He concluded that PK study needs to be done in metronomic chemotherapy trials as they are very informative and because PK parameters could be used as predictive indicators of efficacy. Plasma VEGF and VEGF gene polymorphisms may be a starting point but more mechanisms are involved and need to be explored. For the future, there is a need of a therapeutic drug monitoring of metronomic chemotherapy (to document Css) and a need of PK-PD models.

Nicolas André (Metronomics Global Health Initiative; Aix Marseille Université, Inserm, CRO2 UMR_S 911; & Paediatric Haematology and Oncology Department, Children’s Hospital of La Timone, Marseille, France) introduced the next two speakers, Alexandru Eniu of Romania and Shripad Banavali of India, and the topic of their talk was the use of metronomics (metronomic chemotherapy and drug repurposing) in LMICs [30]. As we enter the 21st century, cancer has become a global disease. The challenges for good cancer care in LMICs are numerous, and he illustrated this with the horrible fact that, as a child with cancer living in one of the 25% poorest countries in the world, you have 90% risk of dying of the cancer. Metronomics should be seen as a chance to come with new innovative affordable approaches and not as a cheap rescue strategy.

Alexandru Eniu (Cancer Institute ‘I.Chiricuta’, Cluj-Napoca, Romania) explained he had limited experience with metronomic chemotherapy but that his interest stems from the Breast Health Global Initiative (BGHI), an initiative which started because the current guidelines [e.g., Europena Society of Medical Oncology (ESMO) or National Comprehensice Cancer Network (NCCN)] cannot be implemented as such in limited resource countries, and therefore other guidelines were required. He underlined that a clinician in those countries is a manager of scarce resources. The BGHI publishes guidelines and organises global summits. It soon found that there is no one size that fits all (heterogeneity) with requirement for resource stratification and proposed guidelines with four levels.

- Basic level are core resources (e.g., operating room).
- Limited level are second-tier resources.
- Enhanced level are third tier resources.
- Maximum level corresponds to what is available in rich countries.

He then illustrated the situation in Ghana, where chemotherapy and hormone therapy could be provided in Kumasi teaching hospital but that the resources were extremely limited in the countryside. They realised that some of the core issues were the absence of medical oncology specialists; the choice of drugs there was not driven by indication but by availability.

Moving to the metronomic approach, he listed the advantages of metronomic treatment in these limited resources countries:

- lower toxicity and therefore better compliance, less frequent visits, potentially fewer blood tests, and no special equipment for following toxicities
- oral treatment, so no need of infusion facilities and possible in a distributed health care system but issues in ensuring compliance
- lower cost, so less cost for the patient and better compliance.

It does not mean there are no issues—for instance, vinorelbine needs to be stored in a cold place.

He also mentioned the importance of the task force working on adding some chemotherapy and hormone therapy drugs on the World Health Organisation (WHO) essential medicines list to increase the number of possibilities. Europe is being affected by a drug shortage of older drugs, as indicated by a recent survey that they carried out and which will be presented at the ESMO conference in September 2014 in Madrid.

He suggested to set up a Global Metronomic Fund which could fund research but also support accessibility of drugs, stating, for instance, that one year of cyclophosphamide-methotrexate maintenance costs 12 euro in Romania.

The next speaker was Shripad Banavali of the Tata Memorial Centre in Mumbai, India, who talked about the Indian experience with metronomic chemotherapy.

He started by showing that using the Maximum Tolerated Dose (MTD) based chemotherapy strategy though we have improved outcome in some solid tumours, for many others there is minimal improvement if at all. Even though the paradigm is changing with the advent of molecular understanding of various cancers and development of targeted therapies, many of the responses are short lasting, and the cost of such treatment is out of reach for most patients in LMICs. He showed that in a large cohort of HER2+ breast cancer patients eligible for trastuzumab at their centre, only 4% of non-trial patients received trastuzumab, the majority of them paying from their pocket [31]. Compared to the situation in Mumbai, the situation in the rural outreach centre of Tata Memorial in Dervan (www.walawalkarhospital.com) is typical of the rural challenge in India. Issues are not only financial but also clinical, as patients present with poor nutritional status, advanced disease, and there is limited infrastructure to give standard therapies. Metronomic chemotherapy is therefore of the highest interest in this type of region. The interest of metronomic chemotherapy is especially interesting in three different clinical settings: relapse or recurrence; peri-operative; and in the maintenance setting. As examples in these settings respectively, he reported of their experience in sarcoma, head and neck (H&N) cancers, and acute myelogenous leukaemia (AML).

In relapsed, refractory, and/or metastatic Ewing and rhabdomyosarcoma, the combination of oral tamoxifen, etoposide, and cyclophosphamide (which costs less than 25 USD a month) gave durable responses. Compared to the literature, the 49 patients had favourable OS of more than 50% at five years [30].

In H&N cancers, the combination of methotrexate and celecoxib (which cost less than 15 USD a month) was associated with increased survival when compared to matched controls in patients with advanced, operable H&N cancers [32].

Finally, in AML, after induction and consolidation, patients received a maintenance treatment with oral etoposide and 6-thioguanine for six months. The relapse rate of 25% compared favourable to historical controls where it was around 45%. Out of the 18 patients who relapsed, 12 patients relapsed within six months of stopping therapy, which suggest that 12 months oral maintenance may prevent some of these relapses. In comparison with results from other groups, the disease free survival is similar to what has been observed in high-income countries using chemotherapy as well as autologous stem cell transplant (ASCT) where indicated.

Nicolas André (Metronomics Global Health Initiative; Aix Marseille Université, Inserm, CRO2 UMR_S 911; & Paediatric Haematology and Oncology department, Children’s Hospital of La Timone, Marseille, France) concluded this part on the LMIC by reporting his experience of collaboration with investigators in LMIC in the framework of the Metronomics Global Health Initiative which was launched to create a worldwide network and support the implementation of trials with metronomics approaches.

In the Metro Mali-01 trial, a vincristine/cyclophosphamide/methotrexate regimen was given to children with refractory cancer of various tumour types. None of the patients experienced any response, but seven out of the 12 patients treated had stable disease, and three of them had a long stabilisation after study discontinuation [33].

The Metro Mali-02 protocol added valproic acid to this regimen and two partial responses were noted out of the seven patients [34]. The metronomic approach is still used in Mali by the investigator, with 52 patients now treated in total.

He also mentioned two other initiatives recently started in Central America and Morocco. In Dominican Republic, Dr Wendy Gómez García from the Robert Reid Cabral Children’s Hospital launched a palliative care pilot programme which includes a metronomic treatment. 29 patients have been included in the metronomic treatment with an ibuprofen–cyclophosphamide-methotrexate protocol. In Morocco Pr Maria El Kababri and Pr Laila Hessissen initiated a metronomic phase II trial with a cyclophosphamide, etoposide, and valproic acid combination in 18 cohorts of children with either a relapsing/refractory tumours or a very advanced disease. In France, a trial in children with low-grade optic pathway gliomas tests fluvastatin with celecoxib (NCT02115074) based on preliminary and preclinical evidence [35].

In the first part of his talk, Eddy Pasquier from the Children’s Cancer Institute, Sydney, Australia presented findings from his lab with the use of β-blockers in cancer treatment. His work showed that β-blockers were able to increase the anti-angiogenic and anti-tumour activity of chemotherapy *in vitro* and in animal models of TNBC [36] and NB [37]. In particular, strong synergism was observed between β-blockers and microtubule-targeting drugs, such as paclitaxel and vincristine.

Two other groups recently confirmed the potential of β-blockers in the treatment of NB [38, 39].

In angiosarcoma, bevacizumab and anti-angiogenic TKI have counter-intuitively repeatedly failed, and more effective treatments are urgently needed. Starting with immortalised and transformed endothelial cell lines, Eddy Pasquier and his team found that these cells expressed high levels of β1- and β2-adrenoreceptors and that propranolol, a non-selective β-blocker, could inhibit their proliferation *in vitro*. Propranolol interacted synergistically with vinblastine against these cell lines, but not with chemotherapeutic drugs commonly used in the treatment of angiosarcoma such as paclitaxel and doxorubicin. The synergism between propranolol and vinblastine was further confirmed in tumour spheroids. Interestingly, five patients with relapse or metastatic angiosarcoma have been treated by Dr Shripad Banavali at the Tata Memorial Centre, Mumbai, with a metronomic combination of propranolol, vinblastine, and methotrexate regimen and all have responded to it. Images of one of these five patients were presented. This patient had a multi-focal angiosarcoma and had a dramatic response, which lasted two years, under this experimental regimen. The plan is to start a clinical trial as soon as possible.

The second part of Eddy Pasquier’s talk was about ‘next-generation’ drug repositioning. His idea is that the use of old drugs for cancer treatment is not sexy enough to attract competitive funding. He proposes an unconventional approach for drug development that relies on high-throughput screening of approved drugs and siRNA. It is well known that drug repositioning can shorten the development time, reduce the cost and lower the risk of failure. He carried out a high-throughput screening of 3600 active compounds screened for NB and found 68 hits that were able to re-sensitise drug-resistant cells to chemotherapy. Bioinformatics analysis then led to the identification of 235 genes potentially involved in drug resistance in NB, 84 of which correlated significantly with patient survival. He is now continuing this work, extending his original methodology to other refractory cancers, and will do it in Marseille, France, as part of the Marie Curie fellowship scheme from the European Research Council.

Francesco Bertolini concluded this session by explaining the recent interest of their lab to look into old drugs. The main problem to him in drug development is that, in the past, the agenda of drug development was mainly driven by academia. It is no more the case as the agenda is set by pharmaceuticals. This might explain some of the recent issues including the skyrocketing cost of drugs.

The typical example of an interesting old drug is aspirin. In their own lab, they started looking into metformin as they found that both metformin and phenformin (now withdrawn from the market) were active to induce apoptosis of endothelial progenitor cells and cancer cells. They tested *in vivo* combinations of metformin and phenformin with aspirin and betablockers and found that the effect was synergistic in both immunocompetent and immunosuppressed mice. In these experiments, phenformin was particularly effective.

Metformin is being extensively tested in cancer trials, including in one preoperative trial by their group. Various recent findings indicate that metformin could replace fasting which has been shown to possibly increase the efficacy of chemotherapy, could be beneficial in combination with radiotherapy and would able to increase the lifespan of mice. This confirms the biological potential of metformin and its interest as a drug reposition to treat some cancers.

## Barton Kamen–MGHI Prize

The first Barton Kamen-MGHI Prize has been awarded to Prof. Shripad Banavali for his abstract entitled ‘Modified combat (combined oral metronomic biodifferentiating antiangiogenic treatment) therapy in poor prognosis paediatric malignant brain tumours—is there a role?’. The prize was given by Ruth Kamen, Barton Kamen’s wife, to Shripad Banavali after a very touching speech in memory of the late Barton Kamen, a paediatric oncologist who pioneered low-dose chemotherapy and was an untiring advocate of metronomic chemotherapy.

Shripad Banavali talked about the modified COMBAT protocol that his group tested in a clinical trial with embryonal tumours with known poor outcomes. They wanted to assess the role of COMBAT in high risk CNS tumours, in comparison with the outcomes reported by Peyrl in 2012 [40] where they used an intensive protocol with rather high toxicity and no dramatic clinical improvement. As opposed to the original COMBAT protocol [41], in the modified COMBAT protocol celecoxib was replaced with sodium valproate considering the importance of epigenetic changes associated with most CNS tumours. A retrospective analysis was done of all patients with relapsed medulloblastomas (MB), relapsed primitive nectodermal tumours (PNETs), relapsed ependymoma, and upfront diffuse pontine glioma, and after amendment upfront high-risk MB and PNET who had received this protocol. For the 51 patients that have been treated, the median age was nine years, and the median number of cycles received was two. Grade 3 toxicity was mainly stomatitis and cheilitis. The median time to progression (TTP) was nine months for the entire group, and the two-year PFS and OS were 32.2% and 55%, respectively. The outcome was significantly better for patients who took at least three months of treatment (two-year PFS 40.7%) and those who had CR or partial remission (PR)—as opposed to gross residual disease—at time of starting modified COMBAT protocol (two-year PFS 50.0%). These outcomes compared favourably with Peyrl’s series. It can also be considered cost-effective, with a cost per cycle of 102 USD compared to 5948 USD (excluding cost of intrathecal drugs) for Peyrl’s protocol. These data helped to generate the hypothesis, whether there is a role for such metronomic maintenance therapy for all patients with high risk embryonal brain tumours, which they plan to prospectively study.

## Session 4: More types of cancer and selected presentations

Martin Villalba (INSERM U1040, Université de Montpellier 1, UFR Médecine & Institute for Regenerative Medicine and Biotherapy, CHU Montpellier, Montpellier, France) focused on tailored metronomic approaches for acute leukaemia. It could be surprising at first sight to use metronomic approaches in acute leukaemia as angiogenesis is not really of importance in leukaemia. But the metronomic schedule could be of interest to act on leukaemia cells with compounds exerting their effect via other angles.

He reported the use of dichloroacetate (DCA) in their lab which acts on the Warburg effect described for almost all cancer cells. However, tumour cell metabolism is not homogeneous. Cancer cells will change the metabolism on which they rely over time because of external factors and conditions. DCA is known to inhibit the enzyme pyruvate dehydrogenase kinase1 (PDK1), a kinase enzyme which inactivates the enzyme pyruvate dehydrogenase (PDH) by phosphorylation. PDH converts pyruvate (a product of glycolysis in the cytosol) to acetyl-coA, which is then oxidised in the mitochondria to produce energy, in the citric acid cycle. By inhibiting PDK1, DCA increases the oxidation of pyruvate in mitochondria and decreases the conversion of pyruvate to lactate. *In vitro*, DCA is able to stop cell proliferation, but it actually does not kill the cells and act via a p53 dependent mechanism. DCA was able to cooperate with p53 activating drugs such as doxorubicin in cells with wild-type p53. This interesting approach is currently being tested *in vivo* in the lab of Francesco Bertolini.

He also provided data indicating that DCA was inducing major histocompatibility complex (MHC) class I expression in cancer cells [42]. Their work also suggests that combining DCA or metformin could improve the immune response induced by immune cell transfer, e.g., NK cells or T cells. Metronomic chemotherapy could also increase this immune response [43].

Nicola Fazio (Unit of Gastrointestinal and Neuroendocrine Tumours, European Institute of Oncology, Milan, Italy) shared his experience of metronomic chemotherapy in low-/intermediate-grade neuroendocrine tumours (NETs) [44]. In patients with metastatic low-/intermediate-grade NET with a high tumour burden, where the therapeutic goal is to stabilise the tumour and ensure good quality of life with a sequence of medical therapies, possibly with low toxicity, a metronomic schedule could be interesting. He could identify only three published clinical trials in NET with metronomic chemotherapy. Brizzi [45] reported the use of 5-FU combined with octreotide long acting repeatable (LAR) in a phase II study of 29 patients with metastatic or locally advanced well-differentiated NET from different primary sites. The metronomic schedule of 5-FU was 200 mg/m2 per day as a intravenous protracted continuous infusion through an elastomeric pump. Four patients experienced at least one grade 3 adverse event. The median TTP of all 29 patients was 22.6 months (range, 2.7–68.5), while the median OS was not reached.There was no significant decrease in circulating vascular epithelial growth factor (VEGF) over time.

The same group reported in 2014 results with another Italian multicentric phase II trial of 45 patients with NETs from mixed primary sites [46], this time with metronomic capecitabine and bevacizumab combined with octreotide LAR. Capecitabine was given orally at 200 mg/day continuously. A 18% PR and 64% stable disease (SD) were observed. Grade 3 toxicities included hand/foot syndrome (11%), proteinuria (4%), and renal toxicity (2%). Proteinuria (all grades) was correlated with longer PFS (p = 0.017). VEGF polymorphisms were not associated with patient outcome.

Koumarianou reported the use of metronomic temozolomide with octreotide LAR and bevacizumab [47] in a consecutive series of 15 patients. Temozolomide was given orally at 100 mg/day continuously. In other tumours, such as glioblastoma multiforme, metronomic schedule of temozolomide has been reported to overcome O-6-methylguanine-DNA methyltransferase (MGMT)-related resistance. In Koumarianou’s study, an impressive 64% response rate (PR+CR) was observed, with a nine-month TTP (range 2–15).

In IEO’s experience, more than 70 patients have been treated with metronomic capecitabine so far, and very few with metronomic temozolomide (unpublished data). Among them metronomic capecitabine has been combined as radio-sensitising with peptide receptor radionuclide therapy (PRRT) with ^177^Lutetium in around 20 patients (unpublished data).

Based on the aforementioned data in the AIOM/ITANET 2013 Italian guidelines for the management of gastroenteropancreatic neuroendocrine tumors (GEP NETs), metronomic chemotherapy has been included as an option (among others) for advanced well-differentiated slow-growing GEP NET with a D (low) level of evidence.

Alfredo Berruti (Department of Medical and Surgical Specialties, Radiological Sciencesand Public Health, University of Brescia, Azienda Ospedaliera Spedali Civili, Brescia, Italy) presented a summary of the potential of metronomic chemotherapy for adrenocortical cancer. In this rare cancer, mitotane is the standard treatment for advanced disease and is increasingly used in adjuvant setting after his group reported in 2007 a benefit in recurrence-free survival with mitotane in patients radically operated [48]. In case of metastatic disease or relapse the EDP-M regimen (etoposide, doxorubicin, cisplatin and mitotane) is also administered but OS remains very low. Metronomic regimen seems of interest as demonstrated in the 46% clinical benefit rate (including 1CR and 1PR) in a trial using gemcitabine together with 5-FU in continuous infusion or with oral capecitabine [49]. Alfredo Berrutialso mentioned three case reports with benefit after multiple lines of chemotherapy. The question that comes to mind in these cases is: why did a metronomic regimen work in heavily pretreated patients? First, patients who did receive four or five lines and are still eligible for another line of systemic treatment have a more indolent disease than the patients who cannot make it until the fourth or the fifth line. Second, the use of sequential lines of chemotherapy with a metronomic schedule may be a better option for patients with advanced disease.

With respect to the potentiating effect of anti-angiogenic agents added to metronomic chemotherapy, he concluded on a note of caution regarding the translation from preclinical results to clinical results as their group found promising *in vitro* results with paclitaxel and sorafenib that could not be confirmed in a clinical trial [50] in which patients even seemed to have accelerated progression with this combination.

Ugo Cavallaro of the European Institute of Oncology, Milan, Italy presented findings from his lab suggesting potential new players and targets in tumour vasculature. After going through the concept of normalisation of the tumour vascularisation as a possible way to affect tumours, he listed the plethora of possible players at the molecular level, VEGF on top of this list.

In their lab, they made the hypothesis that Neural Immunoglobulin-like Cell Adhesion Molecules (Neural Ig-CAM), initially identified in the central nervous system, could have a role in tumour vascularisation. They focused on L1, a prototypic member of the neural Ig-CAM family, which is also expressed in non-neural tissue, for example in immune cells such as dendritic cells where they favour intra- and extravasation. L1 is dysregulated in some tumours and is for instance involved in chemoresistance in ovarian cancer cells. Unexpectedly, L1 was found to be aberrantly expressed in cancer and inflammatory vasculature [51] and its expression was induced by pro-angiogenic and inflammatory cytokines.

Combining a pancreatic cancer mouse model with the specific ablation of L1 in the vasculature, Cavallaro *et al* observed that vascular L1 promotes tumour growth and dissemination. Looking at the vessels in the tumour, they concluded that L1 induced an angiogenic phenotype in endothelial cells and regulated vascular permeability and junctional architecture. Furthermore, the ablation of endothelial L1 promoted tumour vessel normalisation. As far as targeting L1 was concerned, the treatment of tumour-bearing mice with an L1-blocking antibody reduced tumour growth and angiogenesis while enhancing vessel normalisation (Magrini *et al*. JCI 2014).

Cavallaro concluded his talk by stressing the possibility that targeting L1 might open new therapeutic perspectives in the context of anti-angiogenic and/or vessel-normalising approaches.

The session continued with seven presentations selected by the scientific committee.

Gauthier Bouche (Anticancer Fund, Strombeek-Bever, Belgium) presented the Repurposing Drugs in Oncology project or ReDO project. The goal of the ReDO project is to summarise the scientific evidence on non-anticancer drugs which have a possible anticancer activity in order to support their use in clinical trials. This project is a collaboration between the Anticancer Fund and Global Cures, two non-profit organisations based in Brussels, Belgium, and Boston, USA respectively. Gauthier Bouche presented the rationale behind the project which could be summarised by the fact that the most interesting drugs are not or limitedly tested in trials whereas they have a rather high level of evidence of activity. The main reason of this discrepancy is the absence of commercial value of these—often generic—drugs. He listed five of them, namely, mebendazole, nitroglycerin, cimetidine, clarithromycin, and itraconazole, which will be the drugs covered in the first five papers to be published in *ecancermedicalscience*, the peer-reviewed scientific journal of *ecancer*. A policy paper [52] and the article reviewing the evidence supporting the potential of mebendazole [53] have been available since July 10 2014. He concluded by listing other potential drugs of interest and emphasising that repurposed drugs are a great untapped source of possible active, safe, and affordable drugs.

O Graciela Scharovsky (National University of Rosario, Argentina) gave a talk on the experience with metronomic chemotherapy for breast cancer in the preclinical and clinical settings. They worked with two murine mammary adenocarcinoma tumour models. Metronomic chemotherapy with cyclophosphamide + celecoxib or cyclophosphamide + doxorubicin inhibited tumour growth, decreased lung metastases, and increased the median survival time, having low toxicity in both tumour models [54]. Also, she reported that the combination of cyclophosphamide and metformin was able to moderately decrease tumour growth and increase the survival of the mice.

The last part of her talk reported a phase II trial they performed in advanced breast cancer patients progressed to standard chemotherapy with a combination of cyclophosphamide and celecoxib. The main responses were prolonged stable disease (40%, 6/15) and one partial response (7%, 1/15), with a clinical benefit of 47%, and median OS of 45 weeks; neither grade III/IV toxicities nor changes in the quality of life were seen [55]. Looking for biomarkers of response, they could find that the baseline values of serum levels of the ratios VEGF/sVEGFR-2 and VEGF/TSP-1 were significantly associated with TTP and could be considered potential predictors of response [56].

Guido Bocci (University of Pisa, Italy) came back to present for the first time the results of a single arm phase II trial of first line docetaxel, prednisone, oral metronomic cyclophosphamide, and celecoxib in metastatic castration-resistant prostate cancer (mCRPC) patients. When designing the trial, they defined success as having 24 or more of the 37 patients free of progression at six months. This was actually the case as 87% of the patients were free of progression at six months with an impressive waterfall plot in terms of response and good toxicity profile. Median PFS was 15 months and median OS was 33 months. Biomarkers associated with response were counter-intuitive as high VEGF as well as basic fibroblast growth factor (bFGF) indicated longer PFS. The article has now been released (http://www.ncbi.nlm.nih.gov/pubmed/25111199).

As a conclusion, this combination is effective as first line treatment in mCRPC patients with a favourable toxicity. A randomised trial of 159 patients (docetaxel+prednisone*vs*.docetaxel+prednisone and metronomic chemotherapy) is now required to increase the level of evidence in favour of this regimen.

Teresa Di Desidero (University of Pisa, Italy) presented results or metronomic capecitabine and cyclophosphamide in colorectal cancer patients. This trial focused on a pharmacokinetic study with elaborated methods (i.e., high-pressure liquid chromatography analyses) to describe the plasma level of capecitabine and its metabolites (5-DFCR, 5-DFUR and finally 5-FU). The maximal concentration of capecitabine, 5-DFCR, 5-DFUR, and finally 5-FU were observed 0.5 hour, 1 hour, 1.5 hours, and 2 hours after ingestion, respectively. 5-FU level was very low compared to the other metabolites which was to be expected as enzymatic conversion from 5-DFUR to 5-FU occurs mainly in the tumour and not in the normal tissues. The clinical results of this trial were a median PFS of 2.1 months and a median OS of 4.6 months. The regimen was well tolerated.

Prof. Shripad Banavali (Mumbai) combined the next two free papers to give his talk titled ‘A prospective randomised phase II study comparing oral metronomic therapy with intravenous chemotherapy in patients with metastatic, relapsed, or inoperable squamous cell carcinoma of the head and neck region (HNSCC)’. This study was done at the Tata Memorial Hospital in Mumbai. HNSCC accounts for 9–10% of all cancers in India and affects mainly people from poor socio-economic background. Nearly two-thirds presented with advanced stages, and 40–60% of these developed recurrences. Cetuximab-based combination palliative therapy, which is standard of care in high-income countries is afforded by only 1–2% of patients in LMICs. Even combination palliative chemotherapy is ill afforded. Thus, with their experience with oral metronomic chemotherapy [57, 58], which costs approximately 10 USD, they undertook this randomised study to compare oral metronomic chemotherapy (Arm B: Methotrexate 15 mg/m2 once a week + celecoxib 200 mg bid everyday) with standard IV single agent cisplatin (Arm A: 75 mg/m2 every 21 days times maximum six cycles). Most of the patients had oral cavity cancers and most had locally relapsed or advanced disease. Also, most had received prior treatment with radiotherapy and/or chemotherapy. The median PFS and OS were 66 and 152 days in Arm A and 101 and 249 in Arm B, respectively, with a p value of 0.014 and 0.02, respectively. Thus the primary endpoint of the study was met with metronomic chemotherapy leading to a 33% improvement in PFS than cisplatin. In addition, metronomic chemotherapy led to an improvement in OS; it also had fewer side effects, with maintenance of QOL. Oral metronomic chemotherapy has thus become a standard of care for patients with metastatic/recurrent HNSCC patients presently at TMH.

The last talk of the meeting was a very interesting paper presented by Prof. Shripad Banavali from TMH, Mumbai, titled ‘Metronomic maintenance therapy prevents relapses in patients with Triple Negative Breast Cancer (TNBC): A retrospective analysis’. This work was done at the BKL Walawalkar Hospital, Dervan, the rural outreach centre of TMH. TNBC occurs in a higher percentage (30%) of patients in LMICs, including India, where disease is seen in younger women and carries a particularly aggressive course. TNBCs have a higher chance of relapse in spite of increased chemosensitivity, and the prognosis is poor, especially for patients who do not achieve a pathological CR (three-year OS of approximately 68%). The TMH team had gained a lot of experience, with good results using metronomic chemotherapy in patients with recurrent/metastatic TNBCs. Thus, considering the poor outcome in TNBCs, at the rural outreach centre, they started giving maintenance therapy to all TNBC patients after standard therapy which included oral cyclophosphamide/doxorubicin/ fluorouracil (CAF) chemotherapy, surgery, +/- radiotherapy. The results presented were of a retrospective analysis of the outcomes of TNBC patients who did not receive metronomic chemotherapy, and of those who received the same. Metronomic chemotherapy consisted of two phases: Phase I was daily oral celecoxib and cyclophosphamide along with weekly cisplatin times 12 weeks; this was followed by phase II, which consisted of daily oral metformin and cyclophosphamide and weekly methotrexate for one year. Of the 53 evaluable TNBC patients, 20 had received no metronomic chemotherapy and 33 received metronomic chemotherapy. At a median follow-up of 36 months, the excellent event-free survival (EFS) and OS for patients receiving metronomic chemotherapy versus those not receiving was 100% and 100% versus 45.6% and 52.6% (p=0.000). Thus, metronomic chemotherapy may negate the poor prognosis of TNBC patients, and they want to test this in a phase III randomised trial.

## Conclusion

The meeting gave new directions for a better understanding of the role of a metronomic schedule in inducing tumour response or stabilisation. Recent data and new research tools will allow researchers to look into the details of the effect of metronomic chemotherapy on the different tumour environment compartments. Another important lesson is the need for PK/PD studies to adjust doses and improve the outcomes.

The meeting also highlighted important general issues. Looking at the impact of the few phase III trials demonstrating the benefit of a metronomic approach, it was admitted that those approaches were barely used in clinical practice. The main reason seems to be the lack of commercial interest. A strong argument in favour of metronomic approaches is their low cost, which represents a major opportunity for national health services in high-income countries as well as LMICs to save money without compromising on efficacy and safety. However, another solution to deal with the issue of practice change is to perform well-designed clinical trials of metronomic and repurposing approaches demonstrating substantial and undisputable improvement, especially in populations with the greatest unmet needs. Metronomics should always be seen as a chance to come up with new innovative affordable approaches and not as a cheap rescue strategy.
